# The Antioxidant and Skin-Brightening Effects of a Novel Caffeic Acid Derivative, Caffeic Acid-3,4-Dihydroxyphenylpropanolester

**DOI:** 10.3390/antiox14070806

**Published:** 2025-06-29

**Authors:** Kyu-lim Kim, Ju-hee Jeon, Yeonjoon Kim, Kyung-Min Lim

**Affiliations:** 1 College of Pharmacy, Graduate School of Pharmaceutical Sciences, Ewha Womans University, Seoul 03760, Republic of Korea; kstarlim@ewha.ac.kr; 2 Graduate Program in Innovative Biomaterials Convergence, Ewha Womans University, Seoul 03760, Republic of Korea; 3 ACTIVON Co., Ltd., Cheongju 28104, Republic of Korea; jhjeon2110@activon.kr; 4 Qmine Co., Ltd., Suwon 16506, Republic of Korea; yeonjoonkim@qmine.co.kr

**Keywords:** caffeic acid, 3,4-dihydroxyphenylpropanolester (3,4-DHPEA), antioxidant activity, skin brightening

## Abstract

Caffeic acid (CA) is a naturally occurring polyphenol antioxidant found in coffee, tea, fruits, and vegetables, known for its strong antioxidant, anti-inflammatory, and anti-aging properties. However, its cosmetic application is limited because of poor dermal absorption due to its high polarity. This study aimed to evaluate the antioxidant and skin-brightening effects of a novel lipophilic CA derivative, CAD (caffeic acid-3,4-dihydroxyphenylpropanolester). CAD was synthesized by conjugating CA with 3,4-DHPEA, a lipophilic antioxidant derived from olive oil. In both DPPH and ABTS assays, CAD exhibited more potent antioxidant activity than CA. In B16F10 melanoma cells, CAD significantly inhibited melanin production without cytotoxicity at concentrations lower than those required for CA. Cellular assays using DCF-DA staining demonstrated that CAD effectively reduced intracellular ROS levels. Mechanistic studies revealed that CAD inhibited tyrosinase activity and downregulated the expression of TYR, TRP-1, and TRP-2. Additionally, CAD suppressed MITF phosphorylation, along with reduced phosphorylation of ERK and JNK, elucidating its anti-melanogenic mechanism. Importantly, CAD showed dose-dependent skin-brightening effects in the 3D human skin model Melanoderm™, as evidenced by increased lightness and histological evaluation. In conclusion, CAD demonstrates strong potential as a safe and effective antioxidant and skin-brightening agent for cosmetic applications.

## 1. Introduction

ROS, reactive oxygen species, are a diverse family of oxidants derived from molecular oxygen. Excessive ROS production can cause adverse effects and cytotoxicity through nonspecific reactions [1]. Antioxidants can eliminate ROS, and their effects are contingent upon the cell penetration, stability, kinetics, and mechanism of ROS scavenging [2]. Antioxidants are available in both synthetic and natural forms. Natural antioxidants used in cosmetics are derived from a variety of sources, including spices, plants, grains, and fruits [3]. Natural antioxidants can be classified into categories such as phenolic compounds, vitamins, and carotenoids. Phenolic compounds range from simple molecules like caffeic acid to complex polyphenols such as tannins and flavonoids [4]. Vitamins, including vitamin E and vitamin C, are widely recognized for their antioxidant properties, while carotenoids like beta-carotene and lycopene also contribute to antioxidant activity [5].

Antioxidants are widely used in industries such as food, cosmetics, pharmaceuticals, and nutritional supplements. Especially, antioxidants such as vitamins C and E are widely used to mitigate the detrimental effects of ROS on the skin, and a variety of antioxidants are being used in cosmetics [6]. These antioxidants are also used for skin-brightening cosmetics, that target the activation of melanocytes. Melanocytes reside in the basal layer of the epidermis [7], and melanin synthesis can be initiated via several stimuli, including UV radiation or α-melanocyte-stimulating hormone (α-MSH). Melanogenesis occurs within the melanosome, a cellular organelle responsible for the synthesis, storage, and transport of melanin. Melanogenic enzymes such as tyrosinase, TRP-1, and TRP-2 located in the membrane of the melanosome are responsible for the synthesis of melanin [8,9,10]. Several natural antioxidant compounds are being utilized as skin-brightening agents. Notable examples include the polyphenolic compounds resveratrol and ferulic acid [11], niacinamide [12], and the carotenoid compound astaxanthin [13]. However, these natural antioxidants generally exhibit weak brightening activity [14] that is attributable to poor cell penetration.

Caffeic acid, a phenolic acid within the polyphenol antioxidant family, exhibits strong antioxidant properties. When used in cosmetics, it helps stimulate collagen synthesis and prevent premature skin aging [15]. Another phenolic compound, 3,4-dihydroxyphenylethanol (3,4-DHPEA), a compound found in virgin olive oil, exhibits strong antioxidant potency and enhances the stability of refined olive oil [16]. However, caffeic acid and 3,4-DHPEA have low lipophilicity (XLogP3, 1.2 and −0.7, respectively), limiting cellular uptake and skin absorption. Indeed, caffeic acid tends to remain in the stratum corneum, with only a minimal fraction reaching the dermis (less than 5%) [17,18]. 3,4-DHPEA may also have limited efficacy due to low skin absorption [19]. Therefore, it is necessary to improve cellular uptake and skin absorption of these water-soluble antioxidants to expect better efficacy.

In this study, we synthesized a conjugated form of caffeic acid and 3,4-DHPEA, caffeic acid-3,4-dihydroxyphenylpropanolester, CAD (first figure in Section 3) with increased lipophilicity (XLogP3, 3.34) in an effort to improve antioxidant activity and skin absorption. We examined the antioxidant and anti-melanogenic activity of CAD and compared with CA. Finally, we explored its applicability to skin-brightening cosmetics using a 3D human-pigmented epidermis skin model, Melanoderm™.

## 2. Materials and Methods

### 2.1. Materials

Caffeic acid+3,4-DHPEA was synthesized in Qmine Co. (Suwon, Republic of Korea). A stock solution of the chemical was made in dimethyl sulfoxide (Sigma-Aldrich, St Louis, MO, USA) at a concentration of 1 mg/mL.

#### 2.1.1. Synthesis of (E)-3-(3,4-Diacetoxyphenyl)Acrylic Acid

**Scheme 1:**
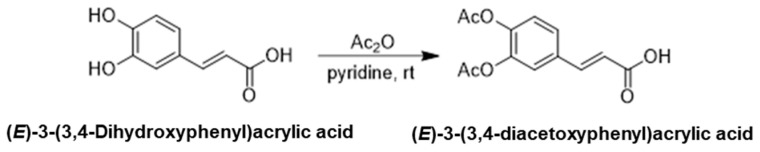
The first step in the synthesis of the target compound: synthesis of (E)-3-(3,4-Diacetoxyphenyl)acrylic acid.

(E)-3-(3,4-Dihydroxyphenyl)acrylic acid(4.5 g, 24.9 mmol) was dissolved in pyridine (6.0 mL, 3.0 eq.) and cooled to 0 °C, treated with acetic anhydride (23.6 mL, 10.0 eq.), which was stirred at room temperature overnight. After the reaction is complete, the reaction mixture was concentrated via rotary evaporation, and the residue was diluted with ethyl acetate and washed with saturated sodium bicarbonate and brine. The organic layer was dried over anhydrous magnesium sulfate and concentrated under reduced pressure. Crude product was purified via silica gel column chromatography to yield (E)-3-(3,4-diacetoxyphenyl)acrylic acid (6.3 g, 95%), (Scheme 1).

#### 2.1.2. Synthesis of (E)-4-(3-(3,4-Dihydroxyphenethoxy)-3-oxoprop-1-en-1-yl)-1,2-Phenylene Diacetate

**Scheme 2:**
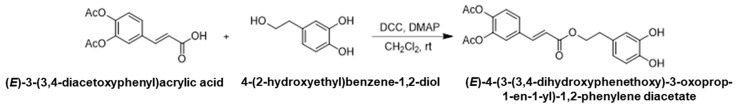
The second step in the synthetic pathway: synthesis of (E)-4-(3-(3,4-Dihydroxyphenethoxy)-3-oxoprop-1-en-1-yl)-1,2-phenylene diacetate.

To a solution of (E)-3-(3,4-diacetoxyphenyl)acrylic acid (13.2 g, 50.0 mmol) and 4-(2-hydroxyethyl)benzene-1,2-diol (8.1 g, 52.5 mmol, 1.05 eq.) in anhydrous dichloromethane (100 mL) under argon atmosphere, N,N-dicyclohexylcarbodiimide (12.4 g, 59.9 mmol, 1.2 eq.) and 4-dimethylaminopyridine (3.1 g, 25.0 mmol, 0.5 eq.) were added. After undergoing stirring at room temperature overnight, the precipitate was filtered off and washed with dichloromethane. The filtrate was collected and washed with 0.1 N hydrochloric acid, saturated sodium bicarbonate and brine. The organic layer was dried over anhydrous magnesium sulfate and concentrated under reduced pressure. A crude product was purified by silica gel column chromatography to give (E)-4-(3-(3,4-dihydroxyphenethoxy)-3-oxoprop-1-en-1-yl)-1,2-phenylene diacetate (5.0 g, 25%) as a white solid, (Scheme 2).

#### 2.1.3. Synthesis of 3,4-Dihydroxyphenethyl (E)-3-(3,4-dihydroxyphenyl)acrylate

**Scheme 3:**
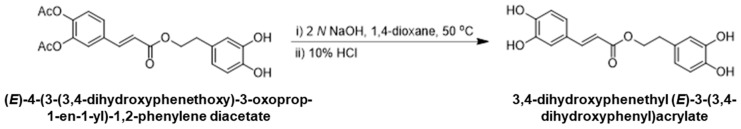
Final step in the synthesis of 3,4-Dihydroxyphenethyl (E)-3-(3,4-Dihydroxyphenyl)acrylate.

To a solution of (E)-4-(3-(3,4-dihydroxyphenethoxy)-3-oxoprop-1-en-1-yl)-1,2-phenylene diacetate (1.0 g, 2.5 mmol) in 1,4-dioxane (10 mL), aqueous 2 N NaOH (3.7 mL, 3.0 eq.) was added and the solution was stirred at 50 °C for 3 h. After cooling to room temperature, 10% HCl (10 mL) and ethyl acetate (15 mL) were added to the reaction mixture and further stirred for 30 min. The organic layer was separated, dried over anhydrous magnesium sulfate, and concentrated under reduced pressure. The residue was purified via silica gel column chromatography with heptane/ethyl acetate as an eluent to provide 3,4-dihydroxyphenethyl (E)-3-(3,4-dihydroxyphenyl)acrylate (0.5 g, 68%). 1H NMR (400 MHz, DMSO-d6): δ 7.46 (d, 1H), 7.06 (s, 1H), 6.81 (d, 1H), 6.65–6.50 (m, 4H), 6.29 (d, 1H), 4.48 (t, 2H), 2.96 (t, 2H). Mass (ESI+): m/z 317.12 (M + H)+, (Scheme 3).

### 2.2. Cell Culture

Urine melanoma B16F10 cells were purchased from ATCC (Manassas, VA, USA) and cultured in Dulbecco’s Modified Eagle’s Medium (DMEM; ATCC) containing 10% fetal bovine serum (FBS; ATCC) and 1% penicillin-streptomycin (Hyclone, South Logan, UT, USA). The cells were maintained every other day and sub-cultured by detaching them using a 0.05% trypsin-EDTA solution (Gibco, Thermo Fisher Scientific, Waltham, MA, USA).

### 2.3. DPPH Assay and ABTS Assay

Additionally,. 100 μM 2,2-diphenyl-1-picrylhydrazyl (DPPH) solution 247.5 μL dissolved in ethanol was mixed with 2.5 μL of the test substance. Ascorbic acid (AA), 10 μg/mL, was used as a positive control. The final treatment concentrations of CA and CAD are 1, 10, 100, and 1000 μg/mL. Experiments were performed in 96-well plates, as described earlier [20]. The samples were added and allowed to react for 0, 15, 30, and 60 min at room temperature in the dark. Then, the absorbance was measured at 517 nm using Infinite M200 Pro microplate reader (Tecan Group Ltd., Mannedorf, Switzerland) equipped at Ewha Drug Development Research Core Center to determine the color change to yellow due to scavenging of free radicals [21]. Ethanol, 250 μL, was used as the blank for the experiment. The data were analyzed using the following equation:

$$\mathrm{Radical}\;\mathrm{Scavenging}\;(\%)=\left\{1-\frac{\left(\mathrm{Abs}\;\mathrm{experiment}-\mathrm{Abs}\;\mathrm{blank}\right)}{\mathrm{Abs}\;\mathrm{Control}}\right\}\times 100$$

A 7 mM 2,2′-azino-bis(3-ethylbenzothiazoline-6-sulfonic acid) (ABTS) solution and a 2.45 mM potassium persulfate solution, both dissolved in PBS, were mixed at a 1:1 ratio in 50 mL conical tube and incubated at room temperature in the dark for 16 h. The following day, the mixture was diluted with PBS to reach an absorbance of 0.700 (±0.002) at 734 nm. Then, 200 μL of the substance was combined with 800 μL of the absorbance-adjusted mixture and allowed to react for 5 min at room temperature. The final treatment concentrations of CA and CAD were 1, 10, 100, and 1000 μg/mL. Experiments were performed in 96-well plates, as described previously [22,23]. The absorbance was measured at 734 nm using a microplate spectrophotometer. PBS 200 μL was used as the blank for the experiment. The data were analyzed using the following equation:

$$\mathrm{ABTS}\;\mathrm{activity}\;(\%)=\left\{1-\left(\frac{\mathrm{Abs}\;\mathrm{experiment}}{\mathrm{Abs}\;\mathrm{Control}}\right)\right\}\times 100$$

### 2.4. Intracellular ROS Level

Intracellular ROS was evaluated using the 2′,7′-dichlorofluoresceindiacetate (DCF-DA, Invitrogen, Eugene, OR, USA) fluorescence staining. DCF-DA was first dissolved in anhydrous DMSO to a concentration of 500 µM and subsequently diluted with PBS to a final concentration of 5 µM. B16F10 cells were seeded at a density of 1 × 10^5^ cells per 2 mL in 35 mm culture dishes. B16F10 cells were treated with 0.2 μM α-melanocyte stimulating hormone (α-MSH) or UVA (1 J/cm^2^), followed by treatment with CA or CAD for 24 h. After incubation, cells were stained with DCF-DA solution for 5 min to assess intracellular ROS levels. UVA irradiation (1 J/cm^2^) was performed using a Biosun UV irradiation system (Vilber Lourmat, Marne-la-Vallée, France). Additionally, 10 μg/mL of AA was used as a positive control. Fluorescently illuminated cell images were taken using the Softmax 5.2 program and an Axiovert 200 M microscope (Carl Zeiss, Land Baden Württemberg, Germany).

### 2.5. Mitochondrial Superoxide Assay

Intracellular mitochondrial superoxide was evaluated via double staining with MitoSOX red (Invitrogen, Eugene, OR, USA) and Mitotracker green (Invitrogen, Eugene, OR, USA). MitoSOX was diluted in anhydrous DMSO to 5 mM and then diluted in Hank’s Buffered Salt Solution (HBSS, Gibco, Thermo Fisher Scientific, Waltham, MA, USA) to a final concentration of 1 μM. Mitotracker green was diluted in anhydrous DMSO to 1 mM and diluted with HBSS to a final concentration of 200 nM. B16F10 cells were seeded at a density of 1 × 10^4^ cells per 400 μL in 4-well culture plates and treated with 0.2 μM α-MSH or UVA (1 J/cm^2^), followed by treatment with CA or CAD for 24 h. Moreover, 10 μg/mL of AA was used as a positive control. After incubation, cells were stained for 30 min and gently washed once with HBSS. The fluorescently stained cells were mounted with fluoromount-G and imaged as a confocal microscope (Zeiss LSM 880 with Airyscan, Carl Zeiss, Oberkochen, Germany) equipped at the Ewha Fluorescence Core Imaging Center.

### 2.6. Melanin Assay and Cell Viability Assay

For melanin content and cell viability measurements, B16F10 cells were seeded at a density of 2 × 10^4^ cells per 600 μL in 48-well plates. After 24 h of seeding, cells were treated for 48 h with CA and CAD in the media containing 0.2 μM α-MSH. Those treated with α-MSH alone were used as the negative control, while 50 μg/mL arbutin-treated cells served as the positive control. To determine melanin content, cells were treated with 40 mg/mL of NaOH solution diluted with 3DW for 1 h and absorbance was measured at 405 nm in a microplate spectrophotometer. Additionally, 40 mg/mL of NaOH solution 100 μL was used as the blank for the experiment. For cell viability determination, cells were treated with 0.2 mg/mL MTT solution diluted in phenol red-free medium for 3 h. After incubation, the medium was removed, and 300 μL of DMSO was added to each well to dissolve the resulting formazan crystals by shaking at 300 rpm for 30 min at 25 °C. The supernatant was then collected, and absorbance was measured at 540 nm using a microplate spectrophotometer. DMSO 150 μL was used as the blank for the experiment.

### 2.7. Mushroom Tyrosinase Inhibitory Assay

To evaluate the inhibition of tyrosinase activity of the substances, the mushroom tyrosinase assay was performed. Experiments were performed in 96-well plates. Moreover, 50 μg/mL of kojic acid (KA) was used as a positive control. The reaction mixture (total volume: 300 μL) consisted of 220 μL of 0.1 M phosphate buffer, 20 μL of test substance (CA or CAD), 20 μL of mushroom tyrosinase (1500 U/mL), and 40 μL of 1.5 mM L-tyrosine. For the blank, 20 μL of DMSO (used as the solvent for the test substances) was added instead of the test substance, and 20 μL of 0.1 M phosphate buffer was used in place of the tyrosinase enzyme solution. The final treatment concentrations of CA and CAD are 1, 10, 100, and 1000 μg/mL. After 15 min of reaction in a dark 37 °C incubator, the absorbance was measured at 490 nm using a microplate spectrophotometer.

### 2.8. mRNA Extraction, cDNA Synthesis, and Real-Time PCR

To analyze the gene expression following CAD treatment, B16F10 cells were seeded at a density of 2 × 10^5^ cells per 2 mL in 6-well plates and treated with CAD for 24 h. After each well was washed with phosphate-buffered saline, Trizol (Invitrogen, Waltham, MA, USA) was added to lyse the cells. Chloroform was added, and the supernatant was collected after centrifugation (13,000 rpm, 15 min, 4 °C). To the supernatant, isopropanol was added, mixed by inverting, and centrifuged (13,000 rpm, 15 min, 4 °C). The isopropanol was carefully removed, and 70% ethanol was added, followed by centrifugation again (13,000 rpm, 5 min, 4 °C). After careful removal of 70% ethanol, the RNA pellet was dissolved in RNase-free diethylpyrocarbonate (DEPC)-treated water. RNA concentration and purity were measured with a nanodrop spectrophotometer (NanoDrop ND1000, Thermo Fisher Scientific, Wilmington, DE, USA). Complementary DNA (cDNA) was synthesized using 1250 ng of RNA and Reverse Transcription Master Premix (Elpis Biotech, Daejeon, Republic of Korea). Relative mRNA expression levels were assessed through real-time PCR, performed with Power SYBR Green PCR Master Mix (Applied Biosystems, Foster City, CA, USA) on a StepOnePlus™ Real-Time PCR System (Applied Biosystems, Foster City, CA, USA). The expression levels of target genes were normalized to β-actin, used as a housekeeping gene. The sequences of the primers were as follows:

Forward β-actin 5′-AGG GAA ATC GTG CGT GAC AT-3′
Reverse β-actin 5′-GGA AAA GAG CCT CAG GGC AT-3′
Forward Tyrosinase 5′-GGG CCC AAA TTG TAC AGA GA-3′
Reverse Tyrosinase 5′-ATG GGT GTT GAC CCA TTG TT-3′
Forward TRP-1 5′-GTT CAA TGG CCA GGT CAG GA-3′
Reverse TRP-1 5′-CAG ACA AGA AGC AAC CCC GA-3′
Forward TRP-2 5′-TTA TAT CCT TCG AAA CCA GGA-3′
Reverse TRP-2 5′-GGG AAT GGA TAT TCC GTC TTA-3′

The following mouse gene accession numbers were used for real-time PCR analysis: β-actin (NM_007393.5), Tyrosinase (NM_001317397.2), TRP-1 (NM_001282014.1), and TRP-2 (NM_010024.3).

### 2.9. Western Blot Analysis

To examine protein expression following CAD treatment, B16F10 cells were seeded at a density of 2 × 10^5^ cells per 2 mL (24 h), 4 × 10^5^ cells per 2 mL (1 h or 4 h), in 6-well plates and treated with CAD for 1, 4, and 24 h. After treatment, a lysis buffer containing RIPA buffer, 1 tablet of protease inhibitor cocktail, and 1 tablet of phosphatase inhibitor cocktail was added to each well. Cells were scraped, and lysates were centrifuged at 12,000 rpm for 10 min at 4 °C. The supernatant was collected, and protein concentrations were determined using the BCA assay. Samples were prepared by mixing a lysis buffer, 4× dye, a 10× reducing agent, and 14 ng of protein, followed by heating at 95 °C. Proteins were separated with 10% sodium dodecyl sulfate-polyacrylamide gel electrophoresis (SDS-PAGE) and transferred to membranes using the Trans-Blot Turbo Transfer System and Trans-Blot Turbo Transfer Pack (Bio-Rad, Hercules, CA, USA). Membranes were blocked with 5% BSA for 2 h at room temperature. The primary antibody against the target protein was bound in 5% BSA overnight at 4 °C. After washing three times with 1× Tris-buffered saline containing 0.1% Tween 20 (TBST), membranes were bound with the secondary antibody in 5% BSA for 1 h at room temperature. After additional washes with TBST, protein detection was performed using the ECL Western Blotting Detection Reagent (Cytiva, Marlborough, MA, USA). Protein bands were visualized using an Amersham Imager 600 (GE Healthcare Life Sciences, Stevenage, UK). BLUEstain™ protein ladder (GoldBio, St. Louis, MO, USA; 11–245 kDa) was used as a molecular size marker. The β-actin antibody (ab8226; Abcam, Cambridge, UK) was used as a housekeeping gene, TYR antibody tyrosinase (ab170905; Abcam, Cambridge, UK), TRP-1 antibody tyrosinase (ab235447; Abcam, Cambridge, UK), TRP-2 antibody tyrosinase (ab221144; Abcam, Cambridge, UK), MITF antibody (ab20663; Abcam, Cambridge, UK), p-MITF antibody (LS-C199259; LSBio, Seattle, WA, USA), p-ERK antibody (4370S; Cell Signaling Technology, Danvers, MA, USA), ERK antibody (4695S; Cell Signaling Technology, Danvers, MA, USA), p-JNK antibody (9251S; Cell Signaling Technology, Danvers, MA, USA), JNK antibody (9252S; Cell Signaling Technology, Danvers, MA, USA), p-p38 antibody (4511S; Cell Signaling Technology, Danvers, MA, USA), and p38 antibody (9212S; Cell Signaling Technology, Danvers, MA, USA) were used.

### 2.10. 3D Human-Pigmented Epidermis Model, Melanoderm™

Melanoderm™ and the EPI-100-NMM-113 maintenance medium were obtained from MatTek Corporation (Ashland, MA, USA). On the day of arrival, tissues were pre-incubated with 5 mL of NMM-113 maintenance medium in a humidified incubator at 37 °C and 5% CO_2_ for 18 h. Following pre-incubation, Melanoderm™ was treated with CA, CAD, and 1% kojic acid (KA) every other day for 14 days, with 1% KA serving as the positive control. Photos were taken before each treatment to assess tissue pigmentation, and ΔL values were measured. After 14 days, tissues were fixed in 10% neutral-buffered formalin and analyzed through Hematoxylin and Eosin (H&E) staining and Fontana-Masson staining.

### 2.11. Statistical Analysis

The data are expressed as means ± standard errors of the means (SEM) obtained from three or four independent experiments. Statistical significance was assessed using Student’s t-test, and a *p*-value of less than 0.05 was considered indicative of a significant difference.

## 3. Results

### 3.1. Antioxidant Effects of CA and CAD in Cell-Free Systems

**Figure 1:**
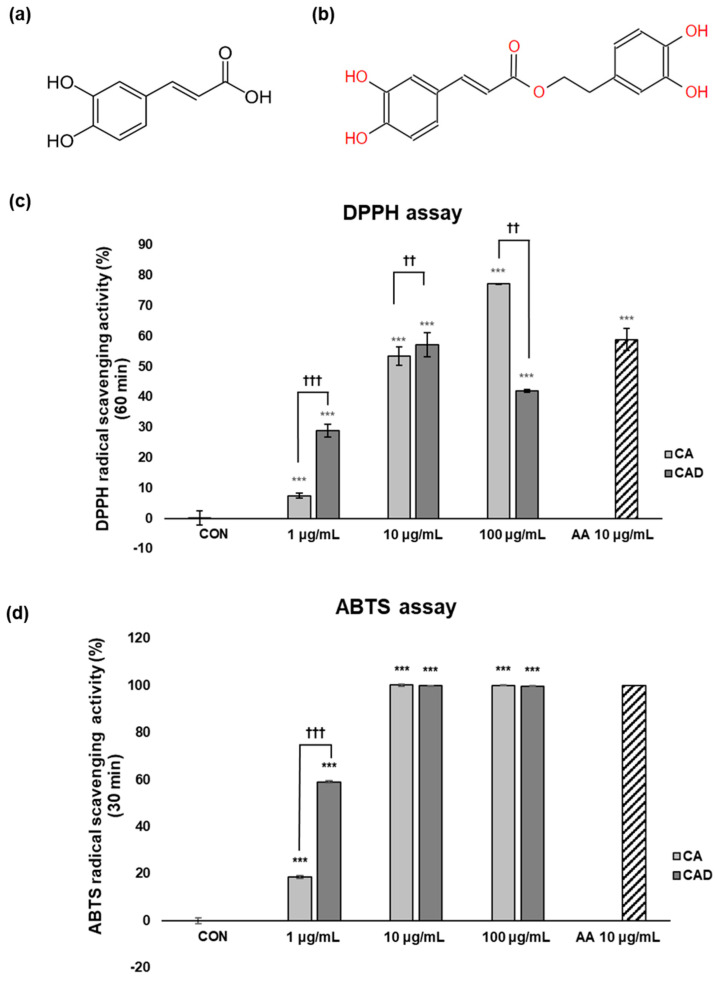
Structures of CA and CAD and their antioxidant activity assessed with DPPH and ABTS assays. Chemical structure of (**a**) caffeic acid (CA) and (**b**) caffeic acid + 3,4-DHPEA (CAD). Antioxidant activity of CA and CAD was measured with (**c**) a DPPH radical scavenging activity assay and (**d**) an ABTS radical scavenging activity assay. In both assays, the percentage of radical scavenging activity was measured at 517 nm. 10 μg/mL of ascorbic acid (AA) was used as a positive control. Data are presented as the means ± SDs (*n* = 4, *** *p* < 0.001 compared with the control, †† *p* < 0.01, ††† *p* < 0.001 comparing CA with CAD).

The antioxidant activities of caffeic acid (CA, Figure 1a) and caffeic acid + 3,4-DHPEA (CAD, Figure 1b) were compared via a DPPH assay and an ABTS assay. The DPPH assay employs a free radical scavenging method and is primarily suitable for evaluating lipophilic antioxidants, whereas the ABTS assay utilizes an anion radical scavenging method and can assess both hydrophilic and lipophilic antioxidants. Conducting both assays together provides complementary results, enabling a more comprehensive and accurate evaluation of antioxidant capacity. As a positive control, 10 μg/mL ascorbic acid (AA) was used. To compare the antioxidant activities of CA and CAD, their radical scavenging activity (RSA%) was evaluated relative to that of the positive control (AA) at the same concentration of 10 μg/mL. In the DPPH assay, the RSA% of CAD was higher than that of CA at 60 min after the reaction at 10 μg/mL (Figure 1c). Similarly, in the ABTS assay, CAD showed higher RSA% than CA at 30 min at 10 μg/mL (Figure 1d). These findings suggest that, in a cell-free system, CAD exhibits more potent antioxidant capacity than CA.

### 3.2. Comparison of Anti-Melanogenesis Effects of CA and CAD in α-MSH Induced B16F10 and Cell-Free Tyrosinase Assay

**Figure 2:**
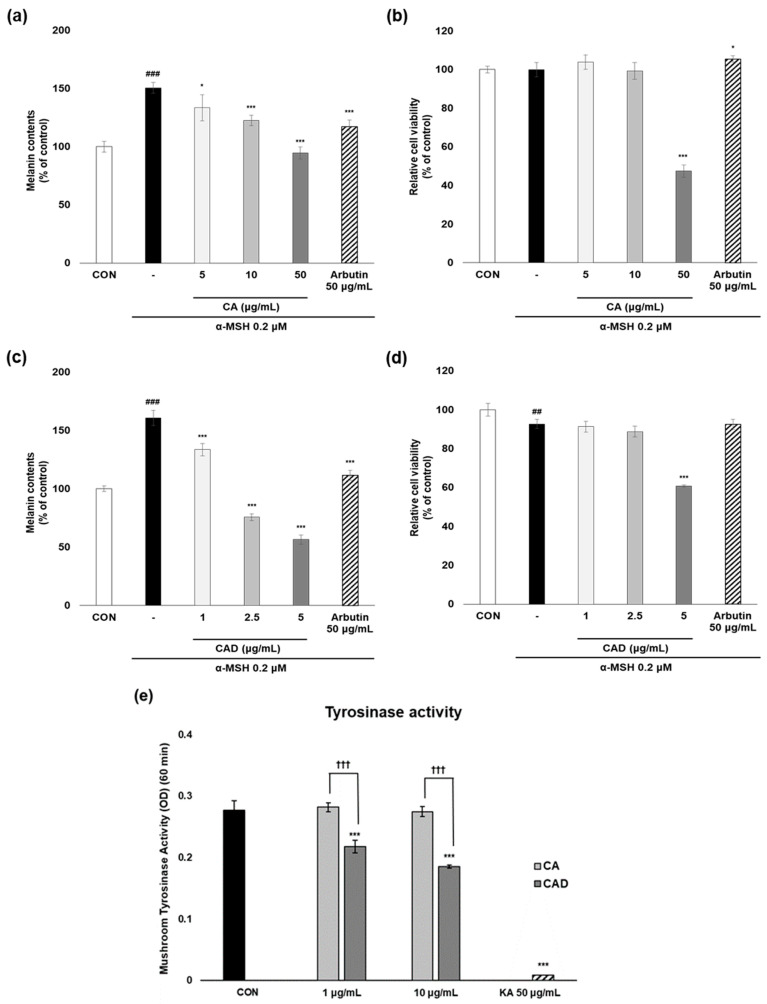
Anti-melanogenesis activities of CA and CAD in cell-based and cell-free system. B16F10 cells stimulated with 0.2 μM α-MSH were treated with CA and CAD for 48 h. (**a**,**c**) Melanin contents were measured using a melanin assay, and (**b**,**d**) cell viability was investigated using an MTT assay. Tyrosinase enzymatic activity in (**e**) CA and CAD was measured using mushroom tyrosinase assay with L-tyrosine as a substrate. Fifty μg/mL of kojic acid (KA) was used as a positive control. Data are presented as the means ± SDs of OD (*n* = 4, * *p* < 0.05, *** *p* < 0.001 compared with the negative control, ## *p* < 0.01, ### *p* < 0.001 compared with the control, ††† *p* < 0.001 comparing CA with CAD).

To evaluate the anti-melanogenesis activity of CA and CAD, melanin contents were measured in B16F10 cells stimulated with α-MSH after treatment with CA or CAD. Arbutin 50 μg/mL was used as a positive control. Following initial experiments conducted across three concentration ranges, two non-cytotoxic concentrations were selected for further analysis, excluding the concentration that exhibited cytotoxicity. As a result, CAD exhibited anti-melanogenesis activity more potent than CA while maintaining cell viability above 80% at the two selected concentrations (Figure 2a–d). The mushroom tyrosinase assay was performed to assess the ability of CA and CAD to directly inhibit tyrosinase activity. While CA failed to inhibit tyrosinase activity up to 10 μg/mL, CAD suppressed it significantly from 1 μg/mL (KA, kojic acid 50 μg/mL as a positive control, Figure 2e).

### 3.3. CAD Reduces Intracellular but Not Mitochondrial ROS Production in B16F10 Stimulated via UVA and α-MSH

**Figure 3:**
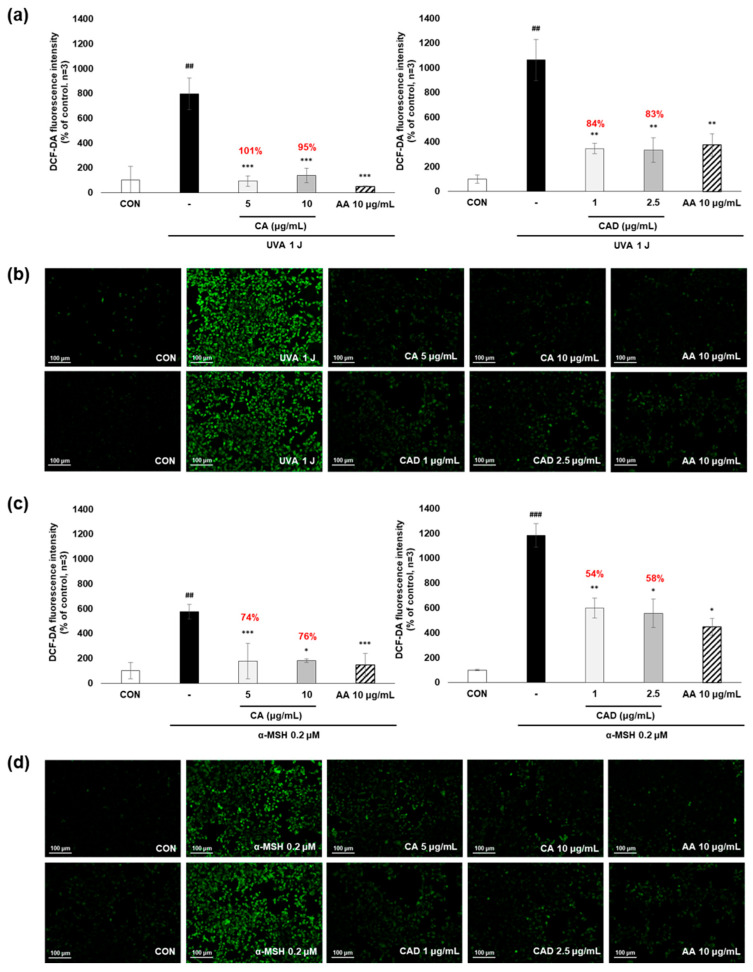

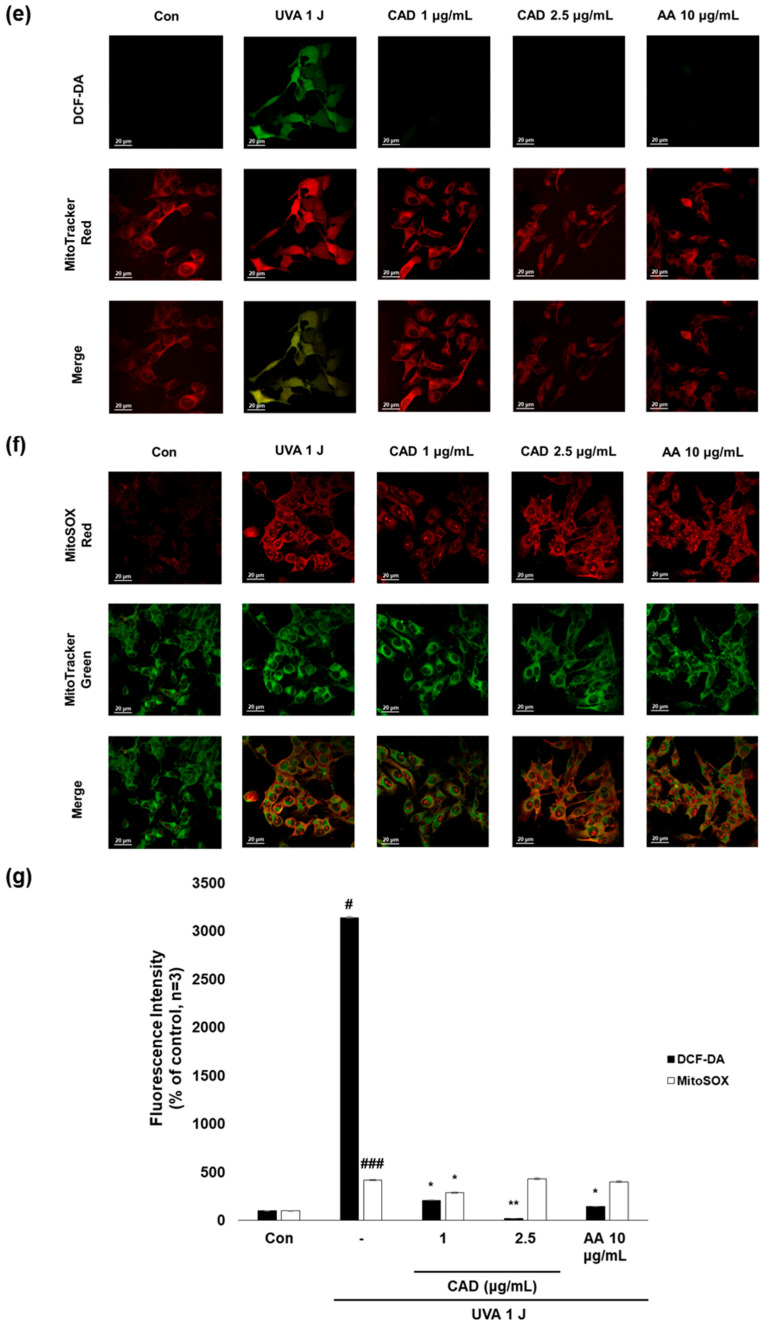

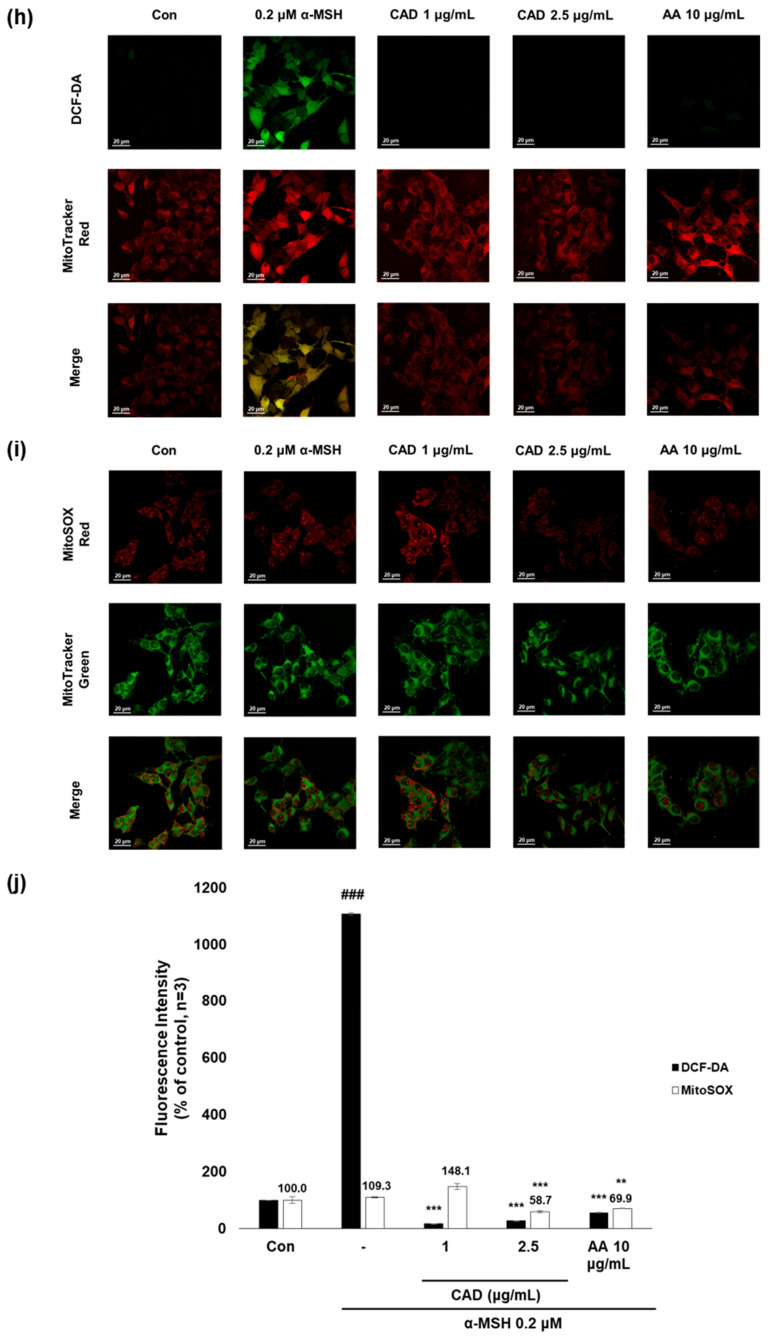
The effects of CA and CAD on ROS production in B16F10 cells induced via α-MSH and UVA. The cells treated with α-MSH and UVA served as the negative control, while those treated with 10 μg/mL AA were used as the positive control. B16F10 cells were irradiated with 1 J UVA or α-MSH at 0.2 μM. CA and CAD were treated for 24 h. (**b**) B16F10 cells stimulated with UVA or (**d**) α-MSH were stained with DCF-DA. (**e**,**f**) B16F10 cells stimulated with UVA or (**h**,**i**) α-MSH were double-stained with DCF-DA green-Mitotracker red and MitoSOX red-Mitotracker green. Representative images were captured via confocal microscopy (400×). (**a**,**c**,**g**,**j**) Fluorescence intensity was analyzed using the Image J software (https://imagej.net/ij/, accessed on 1 September 2023). Data are presented as means ± SDs (*n* = 3, * *p* < 0.05, ** *p* < 0.01, *** *p* < 0.001 compared with the negative control, # *p* < 0.05, ## *p* < 0.01, ### *p* < 0.001 compared with the control).

To evaluate whether CA and CAD can exert antioxidant activities in melanocytes, B16F10 cells, and murine melanoma cells, stimulated with UVA or α-MSH after treatment with CA or CAD and DCF-DA/MitoSOX fluorescence staining was performed. Negative control cells were treated with UVA and α-MSH alone, while positive control cells were treated with 10 μg/mL AA. In all experiments, UVA stimulation induced higher ROS levels than α-MSH stimulation. Fluorescence intensity was quantified using Image J software (https://imagej.net/ij/, accessed on 1 September 2023). To facilitate a quantitative comparison between CA and CAD, ROS inhibition levels were calculated and expressed as a percentage using the following formula: % ROS inhibition = [(UVA or α-MSH-treated (negative control) − CA or CAD-treated)/(UVA or α-MSH-treated (negative control) − untreated control)] × 100, as presented in Figure 3a,c. The DCF-DA fluorescent staining revealed that CA inhibited intracellular ROS generation more effectively than CAD in cells stimulated with either UVA or α-MSH but CAD exerted antioxidant effects at a lower concentration (10 vs. 2 μg/mL) (Figure 3a–d). To further investigate the site of ROS inhibition via CAD, double staining with DCF-DA and Mitotracker red, a mitochondrial dye or MitoSOX red, a mitochondrial ROS-specific dye, and Mitotracker green was conducted. The results indicated that CAD can reduce the total intracellular ROS in the cells stimulated with either UVA or α-MSH, as shown in the merged fluorescence images (Figure 3e,g,h,j). In contrast, mitochondrial ROS levels were not significantly increased via a UVA or α-MSH treatment, and merged fluorescence images confirmed that CAD did not significantly suppress mitochondrial ROS production (Figure 3f,g,i,j). These findings indicate that CAD reduces intracellular ROS levels in cells induced via UVA or α-MSH but could not affect mitochondrial ROS. This localized ROS inhibition of CAD suggests that CAD may modulate oxidative stress through cytosolic pathways without significantly altering mitochondrial ROS production.

### 3.4. CAD Downregulates the Gene and Protein Expression of Melanogenic Enzymes

**Figure 4:**
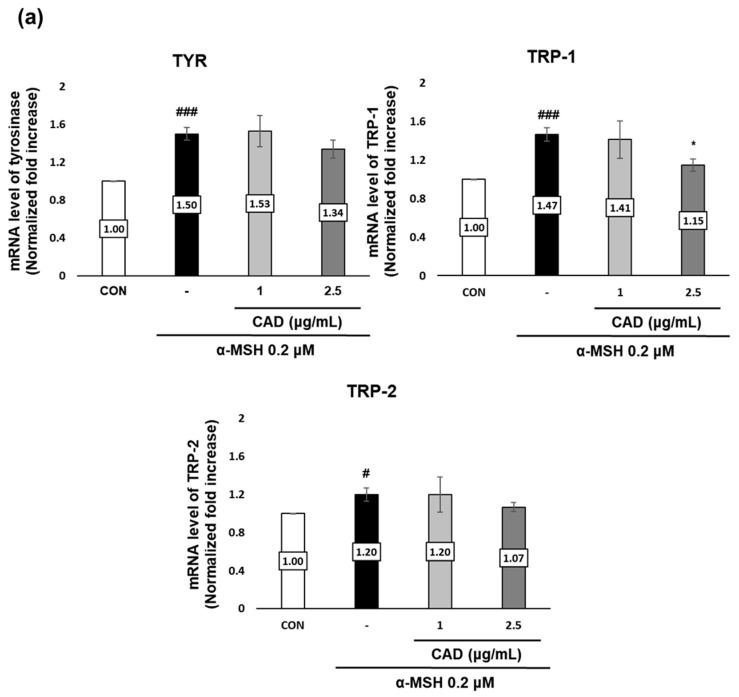

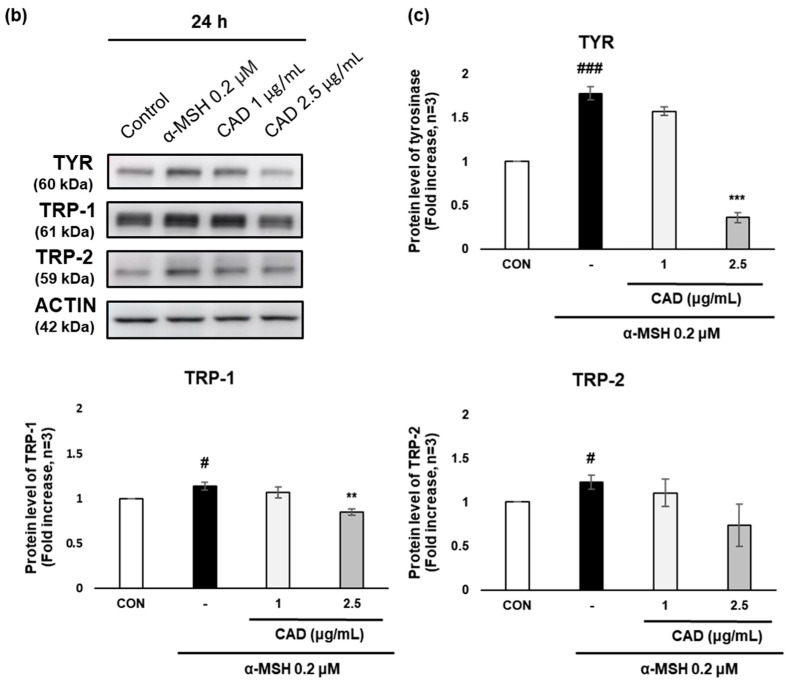
Effects of CAD on mRNA and protein levels of melanogenesis-related molecular targets in B16F10 cells. B16F10 cells stimulated with 0.2 μM of α-MSH were treated with 1 μg/mL and 2.5 μg/mL of CAD for 24 h. Treatment with α-MSH alone served as the negative control. (**a**) mRNA expression levels and (**b**) protein levels of TYR, TRP-1, and TRP-2 in B16F10 cells were analyzed via (**a**) real-time PCR and (**b**) Western blot. (**c**) Quantitation of TYR, TRP-1, and TRP-2 protein levels from the Western blot was performed using the Image J software. For real-time PCR, β-actin was used as the housekeeping gene, and ACTIN was used for Western blot analysis. All data were normalized to the expression of the respective housekeeping genes. Data are presented as the mean ± SD (*n* = 3, * *p* < 0.05, ** *p* < 0.01, *** *p* < 0.001 compared with negative control, # *p* < 0.05, ### *p* < 0.001 compared with control).

The effects of CAD on the gene and protein expression of tyrosinase (TYR), tyrosinase-related protein 1 (TRP-1), and tyrosinase-related protein 2 (TRP-2), key melanogenic enzymes were assessed using real-time PCR and Western blot analysis. Treatment with α-MSH alone significantly upregulated the gene expression of TYR, TRP-1, and TRP-2. In contrast, treatment with CAD significantly downregulated TRP-1 mRNA expression (Figure 4a). A protein expression analysis revealed a significant decrease in TYR and TRP-1 levels and an insignificant but marked decrease in TRP-2 (Figure 4b,c). These findings indicate that CAD inhibits the expression of key melanogenic enzymes.

### 3.5. CAD Inhibits the Phosphorylation of MITF and MAPK Signaling Molecules

**Figure 5:**
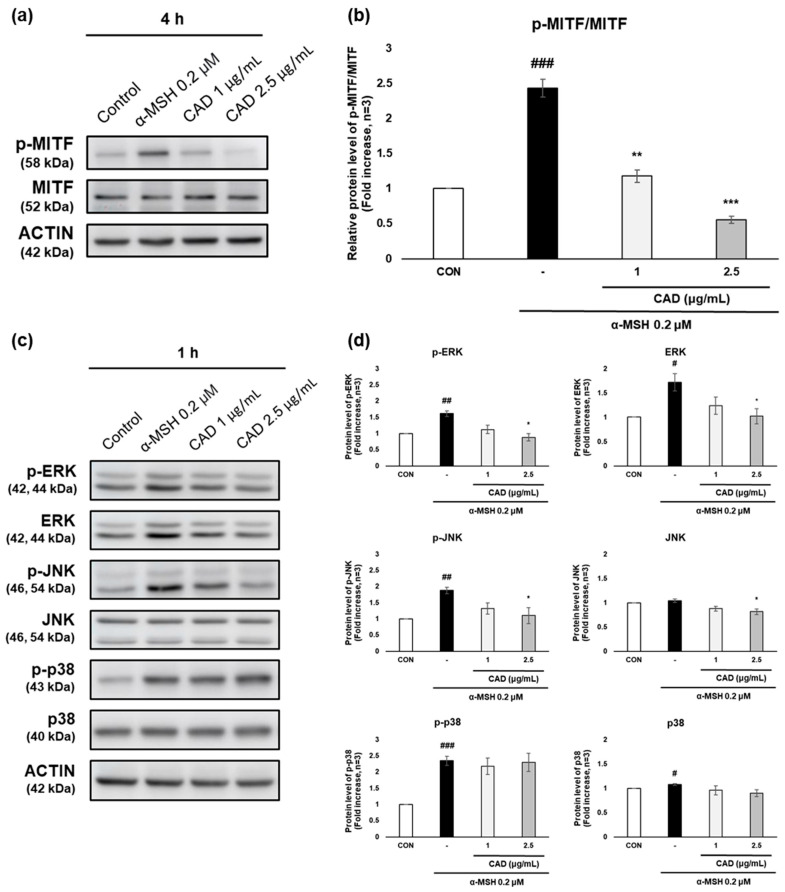
Effects of CAD on phosphorylation of MITF through the MAPK signaling pathway in B16F10 cells. B16F10 cells stimulated with 0.2 μM of α-MSH were treated with 1 μg/mL and 2.5 μg/mL of CAD for 1 h and 4 h. Treatment with α-MSH alone served as the negative control. The protein levels of (**a**) p-MITF and MITF, (**c**) ERK, JNK, p38, and their phosphorylated form were analyzed by Western blot. (**b**,**d**) Quantitation of protein levels from the Western blot was performed using Image J software. (**b**) The data are presented as the comparison of phosphorylated forms to their respective total forms, and (**d**) the data are normalized to the expression of the respective housekeeping genes. Data are expressed as means ± SDs (*n* = 3, * *p* < 0.05, ** *p* < 0.01, *** *p* < 0.001 compared with the negative control, # *p* < 0.05, ## *p* < 0.01, ### *p* < 0.001 compared with the control).

The effects of CAD on MITF, an upstream regulator of TYR, TRP-1, and TRP-2 that are involved in melanocyte stimulation, and on ERK, JNK, and p38, key components of the MAPK signaling pathway, were analyzed. Phospho-MITF (p-MITF) expression increased after 4 h of α-MSH treatment alone but was significantly reduced by CAD. In contrast, the expression of total MITF (MITF) remained unchanged (Figure 5a). As a result, the protein level of p-MITF relative to total MITF was significantly reduced in a concentration-dependent manner via CAD (Figure 5b). Phosphorylation of ERK (p-ERK), JNK (p-JNK), and p38 (p-p38) increased after 1 hr of α-MSH treatment alone. In contrast, CAD significantly reduced p-ERK and p-JNK levels, while it did not significantly affect p-p38, a key factor in the MAPK signaling pathway. The expression of total JNK (JNK) and total p38 (p38) remained stable, whereas the total ERK (ERK) expression followed a similar trend to p-ERK (Figure 5c,d). These findings indicate that CAD effectively inhibits the phosphorylation of MITF and significantly modulates ERK and JNK phosphorylation, key components of the MAPK signaling pathway, contributing to its regulation of melanogenesis.

### 3.6. Brightening Effects of CA and CAD on Melanoderm™, an Artificial 3D Pigmented Human Epidermis Model

**Figure 6:**
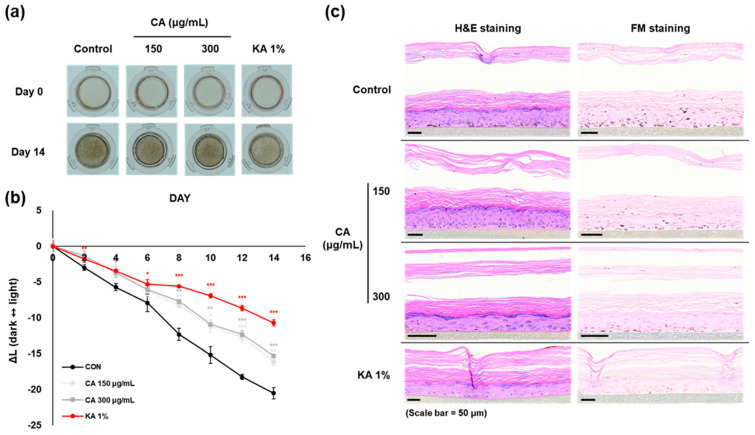

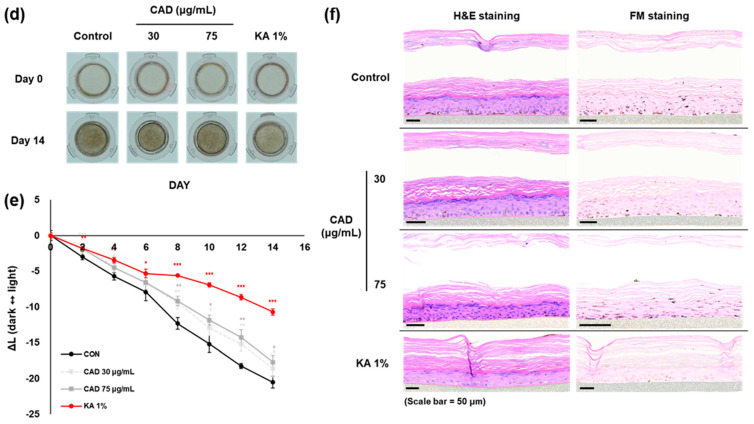
Effects of CAD on Melanoderm™, an artificial 3D pigmented human epidermis model. Melanoderm™, 3D human-pigmented epidermis model was treated with CA 150 μg/mL, 300 μg/mL and CAD 30 μg/mL, 75 μg/mL and KA 1% every other day for 14 days. KA 1% was used as positive control. (**a**,**d**) Observed changes in tissue color on Day 0 and Day 14. (**b**,**e**) The degree of brightness of the tissues was measured every other day as the ∆L-value from Day 0 to Day 14. (**c**,**f**) Tissues on Day 14 were stained with H&E and FM. Data are presented as means ± SDs (*n* = 3, * *p* < 0.05, ** *p* < 0.01, *** *p* < 0.001, compared with the control).

The anti-melanogenic effects of CAD were evaluated in comparison to CA using the 3D pigmented human epidermis model, Melanoderm™. Both CA (150, 300 mg/mL) and CAD (30, 75 mg/mL) were applied every other day, and the tissue brightening was observed on day 14 compared to day 0 (Figure 6a,d). The L-value, representing skin brightness, indicated a reduction in melanin content in both low and high concentrations of CA and CAD (Figure 6b,e). On day 14, skin tissues were fixed and analyzed using Hematoxylin and Eosin (H&E) and Fontana–Masson (FM) staining. The staining results revealed that melanin particles in both CA- and CAD-treated tissues were smaller and more finely distributed compared to the control group (Figure 6c,f). These findings suggest that CAD can manifest skin-brightening effects at concentration ranges than CA.

## 4. Discussion

In this study, we synthesized a novel antioxidant compound, caffeic acid-3,4-dihydroxyphenylpropanol ester (CAD), and investigated its antioxidant and skin-brightening effects. CAD is a derivative of caffeic acid (CA) conjugated with 3,4-dihydroxyphenylethanol (3,4-DHPEA). In silico analysis revealed that CAD had a higher XLogP3 value compared to CA (3.34 vs. 1.2), suggesting greater lipophilicity and potentially improved dermal absorption [24]. Consistent with this prediction, CAD demonstrated significantly stronger antioxidant and anti-melanogenic activities than CA.

Conventional skin-brightening agents such as kojic acid, arbutin, and hydroquinone act primarily through direct inhibition of tyrosinase, a key enzyme in melanogenesis [25]. While CA failed to inhibit tyrosinase activity, CAD significantly inhibited it at 1 μg/mL, indicating that conjugation with 3,4-DHPEA enhances tyrosinase inhibitory potency. In addition, CAD downregulated both mRNA and protein expression of TYR, TRP-1, and TRP-2. Notably, the mRNA level of TRP-1 and the protein levels of TYR and TRP-1 were significantly reduced. The observed discrepancies between mRNA and protein expression may be attributed to differences in the timing of gene transcription versus protein translation and the limited number of sampling time points.

To further elucidate the mechanism underlying CAD’s anti-melanogenic effect, we examined its influence on MITF, the master transcription factor regulating melanocyte function, including differentiation, pigmentation, and survival [26,27], as well as its upstream regulator, the MAPK signaling pathway. CAD significantly suppressed the phosphorylation of MITF, along with that of ERK and JNK—two key components of the MAPK cascade implicated in melanogenic regulation.

Reactive oxygen species (ROS) serve as intracellular signaling molecules, but excessive ROS accumulation induces oxidative stress [28]. ROS can activate the MAPK pathway both directly, via kinases such as apoptosis signal-regulating kinase 1 (ASK1) [29,30], and indirectly, by inactivating phosphatases that dephosphorylate JNK [31,32]. As an antioxidant, CAD effectively reduced intracellular ROS levels, which may contribute to the suppression of ERK and JNK phosphorylation and, consequently, the inhibition of MITF activity and melanogenesis.

The MAPK pathway plays a pivotal role in melanogenesis by regulating MITF. Specifically, phosphorylation of MITF at serine 73 by ERK and JNK enhances its ubiquitination and proteasomal degradation, thereby downregulating the transcription of melanogenic enzymes such as TYR, TRP-1, and TRP-2 [33,34]. Conversely, transient or low-level activation of ERK and JNK may stabilize MITF and promote melanin synthesis. In this study, CAD markedly suppressed ERK and JNK phosphorylation, thereby reducing MITF phosphorylation. This suppression likely underlies the observed downregulation of melanogenic genes and supports the conclusion that CAD mediates its skin-brightening effects through inhibition of the ERK/JNK-MITF axis within the MAPK signaling cascade.

To evaluate CAD’s efficacy in a physiologically relevant system, we utilized a 3D pigmented human epidermis model, Melanoderm™. CAD exhibited skin-brightening effects at both low and high concentrations. An increase in L-value compared to the control indicated reduced melanin content. Histological analysis further confirmed that melanin granules in CAD-treated tissues were smaller and more fragmented. Importantly, CAD demonstrated a brightening effect even at lower concentrations than CA, suggesting its potential effectiveness in human skin applications.

In summary, CAD exhibits strong antioxidant and anti-melanogenic activities, supported by in vitro, in silico, and 3D tissue model data. These findings highlight CAD as a promising candidate for cosmetic applications aimed at skin brightening and antioxidant protection.

## Data Availability

The data presented in this study are available upon request from the corresponding author. The data are not publicly available since they are raw data.
